# MEASUREMENT OF EXERCISE SELF-REGULATORY EFFICACY IN OLDER US VETERANS WITH COPD

**DOI:** 10.1093/geroni/igae098.3487

**Published:** 2024-12-31

**Authors:** Patricia Bamonti, Mariana Wingood, Stephanie Robinson, Grace Rose, Marilyn Moy

**Affiliations:** VA Boston Healthcare System, Boston, Massachusetts, United States; Wake Forest University School of Medicine, Winston-Salem, North Carolina, United States; VA Bedford Healthcare System, Bedford, Massachusetts, United States; VA Boston Healthcare System, Boston, Massachusetts, United States; VA Boston Healthcare System, Boston, Massachusetts, United States

## Abstract

Physical activity promotion and exercise are essential in the management of chronic obstructive pulmonary disease (COPD), a life-limiting disease that causes dyspnea and fatigue. Exercise self-regulatory efficacy is an individual’s confidence to exercise despite barriers and is an essential component to increasing physical activity/exercise. Exercise Self-Regulatory Efficacy scale (Ex-SRES) was developed and initially validated to measure one underlying factor in a small sample of adults with COPD. Research is needed examining the psychometric properties of the scale with larger samples, utilizing objective measures of physical functioning, to further establish its psychometric properties. Secondary analyses were performed on baseline assessment of two cohorts of U.S. Veterans with COPD who participated in a physical activity intervention (n=191; age M =68±8; 97% male;93.2% White). The Ex-SRES asked how confident participants felt they could exercise given 16 unique barriers. Daily steps were assessed for 7-10 days, and exercise endurance was assessed via the 6-minute walk test (6MWT). The St. George Respiratory Questionnaire (SGRQ) assessed health-related quality of life and participants self-reported frequency of days walking for exercise/week. The Ex-SRES demonstrated reliability (Cronbach’s α =.94) and convergent validity: average steps/day (r=.22), 6-MWT distance (r=.25), SGRQ (r=-.20), and walking (r=.30) (p’s<.01). Confirmatory factor analysis demonstrated acceptable fit of a unidimensional factor structure, χ2(458.07, p <.001; SRMR = 0.06; χ2/df = 4.4). Findings are consistent with prior research demonstrating a unidimensional factor structure. However, room for improved model fit is noted. Future research is needed exploring whether a multi-dimensional factor structure may improve fit.

